# Mucoadhesive Chitosome-Based Buccal Delivery of Goat Placenta-Derived Bioactive Macromolecules: Effects on 5-Fluorouracil-Induced Gingival Fibroblast Injury

**DOI:** 10.3390/pharmaceutics18101246

**Published:** 2026-10-01

**Authors:** Worranan Rangsimawong, Phaijit Sritananuwat, Sureewan Duangjit, Natawat Chankana, Praneet Opanasopit

**Affiliations:** 1 Innovation in Drug and Extract of Agriculture Research Center, Faculty of Pharmaceutical Sciences, Ubon Ratchathani University, Ubon Ratchathani 34190, Thailand; phaijit.s@ubu.ac.th (P.S.); sureewan.d@ubu.ac.th (S.D.); 2 Department of Pharmaceutical Chemistry and Technology, Faculty of Pharmaceutical Sciences, Ubon Ratchathani University, Ubon Ratchathani 34190, Thailand; 3 Department of Biopharmacy, Faculty of Pharmaceutical Sciences, Ubon Ratchathani University, Ubon Ratchathani 34190, Thailand; 4 Sun Herb Thai Chinese Manufacturing, College of Pharmacy, Rangsit University, Pathum Thani 12000, Thailand; natawat.c@rsu.ac.th; 5 Department of Industrial Pharmacy, Faculty of Pharmacy, Silpakorn University, Nakhon Pathom 73000, Thailand; opanasopit_p@su.ac.th

**Keywords:** goat placenta extract, gingival fibroblasts, chemotherapy-induced cytotoxicity, chitosomes, mucoadhesive, buccal delivery

## Abstract

**Background/Objectives**: Goat placenta extract (GPE), enriched with bioactive macromolecules, exhibits regenerative potential in both healthy and damaged cells. To enable targeted transport of these macromolecules to the buccal mucosa, potential delivery systems are required. The study aimed to evaluate the bioactivity of GPE on human gingival fibroblasts (HGFs) and to develop a GPE-loaded chitosome (CTS) mucoadhesive system for buccal delivery. **Methods**: The bioactivity of GPE was assessed in normal and chemotherapy-damaged HGF cells. GPE-loaded CTS was prepared, lyophilized, and then incorporated into an oral gel. The *in vitro* buccal deposition was visualized by confocal laser scanning microscopy. **Results**: GPE showed no cytotoxic effects at a concentration of 4000 µg/mL and significantly promoted the viability of both normal and 5-fluorouracil (5-FU)-treated HGF cells (*p* < 0.05). The GPE-loaded CTS formed nanosized vesicles and a positive surface charge, which may facilitate interactions with mucin. CTS and its loaded oral gel enhanced the delivery of macromolecular proteins into the buccal mucosa compared with gel alone. **Conclusions**: GPE promoted HGF viability and facilitated the recovery of 5-FU-induced gingival fibroblast injury. The CTS-loaded oral gel enhanced the buccal delivery of macromolecular protein, representing a promising strategy for oral mucosal regeneration and supportive care in chemotherapy-induced mucosal injury.

## 1. Introduction

Oral mucositis is a frequent and debilitating adverse effect of cancer treatment that significantly affects patient well-being and treatment outcomes. It commonly occurs as a consequence of cytotoxic chemotherapy and radiotherapy, which disrupt the physiological turnover of epithelial cells and compromise mucosal integrity. The inflammatory and ulcerative lesions can markedly diminish patients’ quality of life and may interfere with ongoing cancer treatment. The risk of developing oral mucositis is particularly elevated in patients undergoing radiotherapy for head and neck malignancies, with incidence rates ranging from 30% to 60% [1]. Current understanding of oral mucositis describes its development as a complex biological cascade involving five overlapping stages: initiation, signaling, amplification, ulceration, and healing [2,3]. Palifermin (Kepivance^TM^) is the first FDA-approved agent for preventing and managing oral mucositis, which is a recombinant protein generated from human keratinocyte growth factor (KGF), a member of the fibroblast growth factor family, selectively stimulating epithelial proliferation, tissue repair, and mucosal barrier integrity. Clinically, palifermin reduces the severity of chemo- and radiotherapy-associated toxicities and improves patient quality of life. However, its clinical utility is constrained by protein instability and challenges associated with large-scale production [4,5].

Goat placenta is particularly rich in hydrophilic proteins, amino acids, and growth factors, including insulin-like growth factor-1 (IGF-1), epidermal growth factor (EGF), fibroblast growth factor (FGF), and transforming growth factor-β1 (TGF-β1). These bioactive constituents have been reported to stimulate cellular proliferation, migration, and tissue repair, supporting their potential approach for the management of oral mucositis and other epithelial injuries [6]. Exogenous proteins and growth factors have attracted considerable attention as therapeutic agents due to their crucial roles in cell and tissue repair [7]. In addition, amino acids stimulate fibroblast proliferation and enhance collagen synthesis [8]. Nevertheless, the clinical application of peptide- and protein-based therapeutics via the buccal route is constrained by their poor mucosal permeability. The organized neutral lipid matrix within the superficial epithelial layers acts as a major barrier to macromolecular transport, restricting tissue penetration and limiting therapeutic efficacy. Conventional liquid formulations are also limited by involuntary swallowing and continuous salivary dilution, which compromise residence time and controlled release. Mucoadhesive dosage forms, such as gels, films, tablets, and patches, enhance mucosal contact and local drug retention. Furthermore, incorporation of absorption enhancers or permeabilizers within bioadhesive systems is often necessary to achieve effective peptide and protein delivery [9].

Chitosomes (CTS), also known as chitosan-coated liposomes, are nanovesicles modified by deposition of a chitosan layer on the vesicle surface, which confers a positive surface charge and alters colloidal characteristics [10]. This surface modification facilitates the entrapment of hydrophilic macromolecular proteins within the aqueous core of liposomes and significantly enhances loading capacity [11]. In addition, positively charged chitosan coating markedly improves mucoadhesive performance compared with uncoated liposomes [12].

The combination of liposomal and chitosan gel as a mucoadhesive system enhanced stability, extended residence time, and controlled release of encapsulated bioactive compounds. These properties are particularly advantageous for wound healing and tissue regeneration [13]. However, the efficacy of CTS and its loaded oral gel has not been determined for the delivery of goat placenta extract (GPE) to the buccal mucosa. This study aimed to evaluate the bioactivity of GPE on human gingival fibroblasts (HGFs) and to develop a GPE-loaded CTS mucoadhesive system for buccal delivery. The bioactivity of GPE was assessed in normal and chemotherapy-damaged HGF cells. GPE-loaded CTS was prepared, lyophilized, and then subsequently incorporated into an oral gel. *In vitro* buccal deposition was further evaluated using confocal laser scanning microscopy (CLSM) to visualize the distribution of the formulation within the buccal mucosal tissue. The novelty of this study therefore lies in the application of bioactive GPE to promote the recovery of 5-FU-damaged gingival fibroblasts and the integrated evaluation of this specific GPE-CTS oral gel system for localized buccal delivery.

## 2. Materials and Methods

### 2.1. Materials

Fresh goat placenta was gifted from VR Agro Co., Ltd. (Maha Sarakham, Thailand). HGF cells (ATCC CRL-2014, Lot. 70026474) were purchased from the American Type Culture Collection (ATCC, Manassas, VA, USA). 3-(4,5-Dimethylthiazol-2-yl)-2,5-diphenyltetrazolium bromide (MTT), 5-fluorouracil (5-FU), and bovine serum albumin–fluorescein isothiocyanate conjugate (BSA-FITC; molecular weight of albumin: 66 kDa; molecular weight of FITC: 389.4 Da) were purchased from Sigma-Aldrich (St. Louis, MO, USA). Dulbecco’s modified Eagle’s medium (DMEM), fetal bovine serum (FBS), trypsin–ethylenediaminetetraacetic acid (trypsin–EDTA), GlutaMAX™, non-essential amino acids, and penicillin–streptomycin were purchased from Gibco BRL (Rockville, MD, USA). Phosphatidylcholine (PC; Phospholipon^®^ 90G) was supplied by Lipoid GmbH (Ludwigshafen, Germany), while cholesterol was obtained from Carlo Erba Reagents (Rodano, Italy). Chitosan with a number-averaged molar mass (Mn) of 8000 g/mol, determined by gel permeation chromatography, and a degree of deacetylation of approximately 88%, estimated by nuclear magnetic resonance (NMR) spectroscopy, was purchased from OliZac Technologies Co., Ltd. (Bangkok, Thailand), as reported in the literature [12]. Polyvinylpyrrolidone (PVP K90) was purchased from Namsiang Group (Bangkok, Thailand), and polysorbate 20 (Tween^®^ 20) was purchased from Cheme Cosmetics (Bangkok, Thailand). Lissamine™ rhodamine B 1,2-dihexadecanoyl-sn-glycero-3-phosphoethanolamine triethylammonium salt (Rh-PE) was purchased from Invitrogen (Carlsbad, CA, USA). All other chemicals and reagents were of analytical grade.

### 2.2. Extraction of Goat Placenta

Fresh goat placenta was extracted according to the method reported by Tansathien et al. [6]. In brief, minced placenta and distilled water at a ratio of 1:10 (*w*/*v*) were mixed and probe-sonicated using a Vibra-Cell™ High-Intensity Ultrasonic Processor VCX750 (Sonics & Materials, Inc., Newtown, CT, USA) at a frequency of 20 kHz (20% amplitude) for 30 min under ice bath conditions. The extract was centrifuged at 4000 rpm for 15 min, and the supernatant was collected. The aqueous extract was subsequently freeze-dried at −49 °C for 72 h using a freeze dryer (FreeZone 2.5, Labconco, UK). The dried extract was sterilized by gamma irradiation prior to further analyses, with the specified dose range set at 10–35 kGy and the actual calculated doses received ranging from 12.3–27.5 kGy.

The total protein content of GPE was determined using a bicinchoninic acid (BCA) protein assay kit (Novagen^®^, EMD Millipore Corp., Madison, WI, USA). KGF, a key growth factor involved in epithelial regeneration and wound healing, was selected as a representative bioactive marker and quantified using a commercially available enzyme-linked immunosorbent assay (ELISA) kit (Abcam, Cambridge, UK).

### 2.3. Cytotoxicity Study on HGFCells

HGF cells (10^4^ cells/well) were incubated in a 96-well plate at 37 °C in a humidified atmosphere of 95% air and 5% CO_2_. The cells were maintained in DMEM supplemented with 10% FBS, 1% non-essential amino acids, and 0.1% penicillin–streptomycin. After cell attachment, HGF cells were exposed to varying concentrations of GPE (0–4000 µg/mL) and incubated for 24 h. After incubation with MTT solution (100 µL, 1 mg/mL) for 4 h at 37 °C, the medium was discarded, and cells were washed with phosphate-buffered saline (PBS), pH 7.4. The intracellular formazan crystals were dissolved in dimethyl sulfoxide (DMSO), and the absorbance was measured at 550 nm using a microplate reader (VICTOR Nivo™ Multimode Plate Reader, PerkinElmer, Rodgau, Germany). Cell viability was calculated using Equation (1).

(1) $$\%\mathrm{Cell viability}=\frac{\mathrm{Absorbance of treated cells}}{\mathrm{Absorbance of untreated cells}}\times 100$$

### 2.4. Cytoprotective Effect Assay

HGF cells (5 × 10^3^ cells/well) were cultured in 96-well plates. After incubating overnight, various concentrations of GPE were added to the cells and incubated for 24, 48, or 72 h. Cell viability was assessed using the MTT assay as described in the cytotoxicity study. The percent cell viability was calculated using Equation (1).

To investigate the regenerative effect of GPE on chemotherapy-induced cellular damage, HGF cells were exposed to 5-FU (10 mg/mL) as a chemotherapeutic agent (Figure S1). HGF cells (5 × 10^3^ cells/well) in 96-well plates were subjected to two treatment protocols: pre-treatment and post-treatment. In the pre-treatment protocol, cells were first exposed to GPE for 24 h, after which 5-FU was added without removing the GPE. In the post-treatment protocol, cells were initially exposed to 5-FU for 24 h to induce cellular injury, after which GPE was added without removing the 5-FU. In both protocols, the respective treatments remained in the culture medium throughout the experimental period. Cell viability was evaluated using the MTT assay at 24, 48, and 72 h after the addition of 5-FU in the pre-treatment protocol or after the addition of GPE in the post-treatment protocol. Following MTT treatment, the absorbance was measured using a microplate reader, and cell viability was calculated according to Equation (1).

### 2.5. In Vitro Scratch Assay

The wound-healing potential of GPE was investigated by assessing the migratory activity of HGF cells using an *in vitro* scratch assay. HGF cells (3 × 10^5^ cells/well) were cultured in 6-well plates and allowed to grow as a confluent monolayer. A straight scratch was then generated across the cell layer using a sterile pipette tip. Detached cells and debris were removed by gently rinsing with PBS pH 7.4. The scratched monolayers were subsequently exposed to GPE, 5-FU, or their combinations following the previously defined pre-treatment and post-treatment experimental designs. The control group was the cells maintained without treatment. Wound closure and cell migration were monitored by capturing images at 0, 24, 48, and 72 h.

### 2.6. Formulation of GPE-Loaded Liposomes

Liposomal formulations were prepared using thin film hydration and sonication methods [14]. The liposome compositions are shown in Table 1. Briefly, a lipid mixture composed of PC and cholesterol at a molar ratio of 10:2 mM was dissolved in ethanol (99.8% *v*/*v*) and transferred into a test tube. The solvent was then removed under a gentle nitrogen stream. A thin lipid film was stored in a desiccator overnight to remove any remaining solvent. For the preparation of GPE-loaded liposomes, an aqueous phase containing GPE (0.2% *w*/*v*) and polysorbate 20 (2% *w*/*v*) was used to hydrate the dried lipid film. Probe sonication at 20% amplitude and 20 kHz frequency for 30 min in an ice bath was used to reduce the vesicle size. Excess lipid aggregates were removed by centrifugation at 4000 rpm for 15 min, and the supernatant was defined as conventional liposomes (CL). To obtain CTS formulations, chitosan solution (10 mM in 1% HCl) was incorporated into the liposomes at a volume ratio of 1:9. The mixture was gently stirred for 1 h using a magnetic stirrer to allow surface coating via electrostatic interaction.

Each liposomal formulation was lyophilized in the presence of trehalose as a cryoprotective agent. Preliminary studies showed that a liposome dispersion-to-trehalose ratio of 1:1 (mM) produced a dry powder with acceptable physical characteristics while retaining the characteristic functional groups of GPE, as confirmed by FTIR analysis. Therefore, trehalose was incorporated into the liposomal dispersion at a 1:1 (mM) ratio, followed by freezing at −80 °C for 3 h. The frozen sample was then subjected to lyophilization for 72 h using a freeze dryer. The dried liposomes loaded with bioactive macromolecules were stored at 4 °C until further analysis.

### 2.7. Characterization of GPE-Loaded Liposomal Formulations

Each liposomal formulation was resuspended in deionized (DI) water. The diameter size, polydispersity index (PDI), and zeta potential were determined using dynamic light scattering (DLS) and electrophoretic light scattering techniques with a Zetasizer Nano ZS (Malvern Instruments, Worcestershire, UK) equipped with a 4 mW He–Ne laser (a scattering angle of 173°). Measurements were performed under ambient conditions in triplicate.

Entrapment efficiency was measured using a centrifugal filter tube [15]. Briefly, 2 mL of the liposomal formulation was transferred into an ultrafiltration device (Amicon^®^ Ultra Centrifugal Filter, 100kDa MWCO, Merck Millipore Ltd., Cork, Ireland). The centrifugation was performed at 4000 rpm, 4 °C for 15 min. The liposome-retained fraction was subsequently disrupted with 0.1% Triton™ X-100 at a ratio of 1:1 (*v*/*v*). The protein content was then quantified using a BCA assay kit, and absorbance was measured at 562 nm using a microplate reader. To account for potential matrix interference in the BCA assay, blank CTS without GPE was prepared and subjected to the same centrifugal filtration and protein quantification procedures. The protein signal obtained from the blank formulation was used to correct the measured protein content of the GPE-loaded formulation. The percent entrapment efficiency (%EE) was calculated according to Equation (2).

(2) %EE=Protein entrapped nanocarriers Initial protein loaded×100

Protein recovery of the formulation was determined by disrupting the liposomal vesicles with 0.1% Triton™ X-100 at a 1:1 (*v*/*v*) ratio. The total protein content was subsequently quantified using a BCA assay kit following the procedure described above. The percentage protein recovery was calculated as the measured protein content relative to the initial protein content loaded into the formulation.

Attenuated total reflection–Fourier-transform infrared (ATR-FTIR) spectroscopy (NICOLET 6700, Thermo Electron Corporation, Madison, WI, USA) with the wavenumber range of 4000–400 cm^−1^ was employed to characterize the molecular structure and potential interactions among the formulation components. The resulting spectra were processed and analyzed using OMNIC software version 7.3 (Thermo Electron Corporation, Madison, WI, USA).

### 2.8. Particle–Mucin Interaction of GPE-Loaded Liposomal Formulations

The interaction of liposomes and mucin was measured following the modified method from Forte et al. [16]. Mucin (2 mg/mL) was dissolved in HEPES buffer (pH 6.3–6.7). Equal volumes of the liposomal dispersion and mucin solution were mixed and incubated at 37 °C for 15 min. After that, the particle size and zeta potential of the liposome–mucin complexes were monitored.

### 2.9. Formulation of Oral Gel Containing GPE-Loaded Liposomal Formulations

An oral gel base composed of 5% *w*/*w* PVP K90, 10% *w*/*w* polyethylene glycol 400, 5% *w*/*w* polysorbate 20, 0.1% *w*/*w* menthol, 0.1% *w*/*w* clove oil, 0.4% *w*/*w* peppermint oil, 2% *w*/*w* poloxamer 407, 10% *w*/*w* glycerin, 0.5% *w*/*w* sodium benzoate, 0.1% *w*/*w* potassium sorbate, 0.05% *w*/*w* saccharin, 5% *w*/*w* xylitol, 10% *w*/*w* sorbitol, 0.05% *w*/*w* ethylenediaminetetraacetic acid disodium salt, and purified water (q.s. to 100% *w*/*w*) was used. GPE (0.1% *w*/*w*) with and without a liposomal system was homogeneously mixed with the gel base. The viscosity was measured with a rheometer (Thermo Scientific™ HAAKE™ Mars III, Thermo Scientific, Karlsruhe, Germany) equipped with a C60/1 Ti cone-and-plate geometry. The measuring gap was fixed at 0.1 mm. The test consisted of a temperature equilibration/rest step followed by a logarithmic shear-rate ramp from 0.1 to 1000 s^−1^ at 20 °C. The shear stress and apparent viscosity were recorded as functions of shear rate (Figure S2). To compare the formulations, the apparent viscosity at the midpoint of the flow curve was recorded. The pH of the formulation was measured with a pH meter (SD50, METTLER TOLEDO, Nänikon, Switzerland) at room temperature. The prepared gels were stored at 4 °C before use.

### 2.10. Visualization of Buccal Deposition

Porcine buccal mucosa was obtained from freshly sacrificed pigs at a local market (Ubon Ratchathani, Thailand), with appropriate ethical approval for collection by the Investigational Review Board (34/2568/IACUC, Animal Experimentation Ethics Committee, Ubon Ratchathani University). Mucosa tissues were trimmed off with surgical scissors to remove excess connective and adipose tissues (thickness~0.4–0.7 mm). Generally, proteins and peptides present within the buccal tissue may interfere with the accurate quantification of externally administered macromolecules; BSA-FITC was selected as a fluorescent model macromolecular protein. In addition, Rh-PE-labeled phospholipids were incorporated to trace the nanocarriers [15]. Buccal deposition was evaluated using a vertical Franz diffusion cell with a diffusion area of approximately 2.05 cm^2^. The receptor compartment was filled with 6 mL of PBS pH 7.4 and maintained at 37 ± 2 °C under constant magnetic agitation. Each test formulation (2 mL) was carefully placed on the mucosal surface of the excised buccal tissue. After 4 h of penetration, the tissues were carefully rinsed with distilled water to remove surface-associated formulation.

The localization and penetration of fluorescently labeled compounds within buccal tissue were examined using confocal laser scanning microscopy (Zeiss LSM 800, Carl Zeiss, Jena, Germany) equipped with diode lasers operating at 405, 488, and 561 nm. Imaging was performed with a 10× objective lens. Fluorescence distribution throughout the tissue was semi-quantitatively analyzed using ZEISS ZEN software (version 2.3 SP1), which generated fluorescence intensity profiles along the central horizontal axis of each image. One tissue specimen was analyzed for each formulation. These data were used to construct depth-dependent fluorescence intensity curves, enabling comparison of distribution characteristics among the tested formulations.

### 2.11. Data Analysis

Data are presented as the mean ± standard deviation (SD). All statistical analyses were performed using IBM SPSS Statistics version 27.0 (IBM Corp., Armonk, NY, USA). For the biological studies, three technical replicates from a single independent experiment were performed. At each predefined time point, the experimental data were analyzed independently to assess differences among treatment concentrations and the corresponding control using one-way analysis of variance (ANOVA), followed by Tukey’s multiple-comparison test. A paired *t*-test was used to compare the physicochemical properties of the formulations. Statistical significance was defined as *p* < 0.05.

## 3. Results

### 3.1. Bioactivity of GPE on Gingival Fibroblast Cells

**Figure 1:**
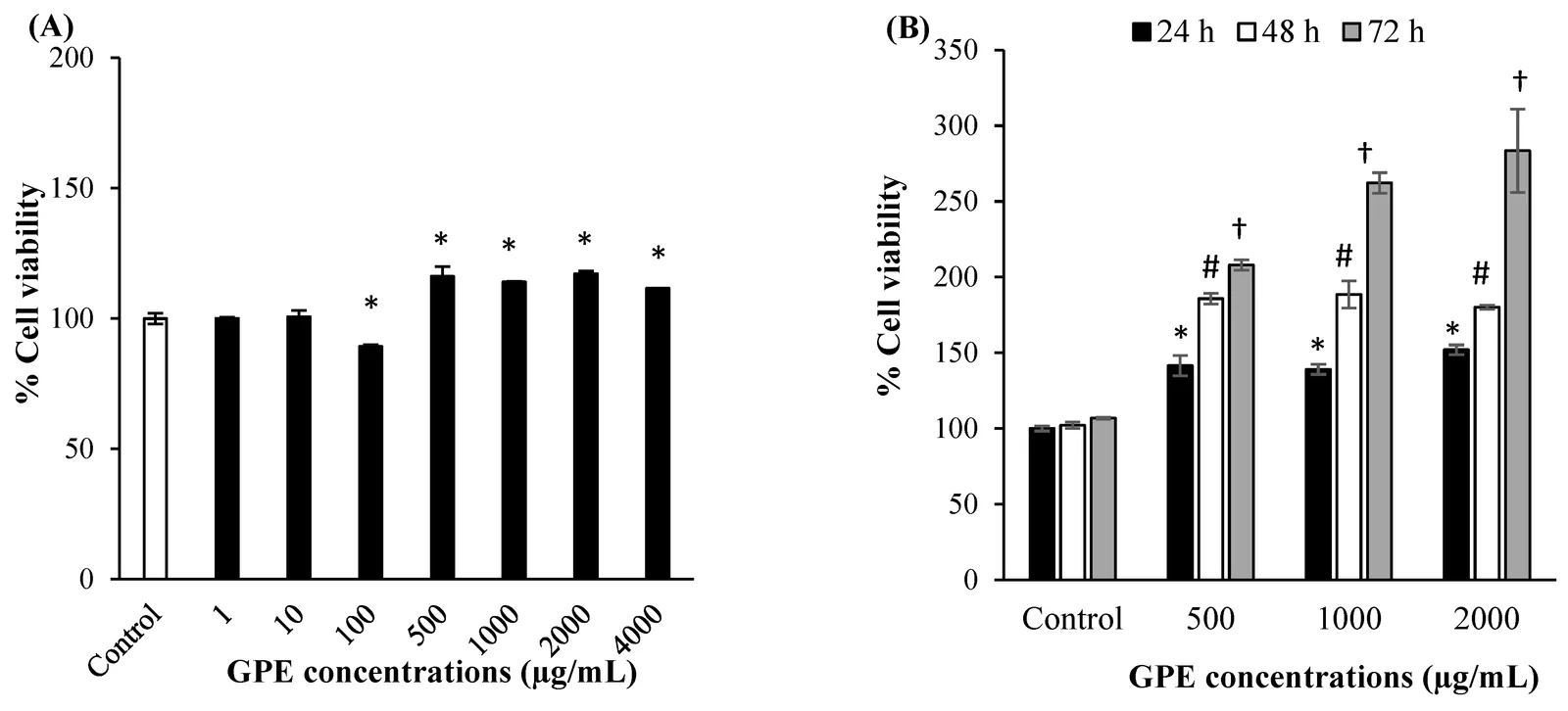
Percent cell viability of HGF cells (10^4^ cells/well) after 24 h (**A**), and HGF cells (5 × 10^3^ cells/well) after 24, 48, and 72 h (**B**), following treatment with various concentrations of GPE. Data are presented as mean ± SD (*n* = 3). Asterisks indicate statistically significant differences compared with the untreated control group at each time point: * at 24 h (*p* < 0.05), # at 48 h (*p* < 0.05), and † at 72 h (*p* < 0.05).

The GPE was rich in bioactive constituents, with a total protein content of 544 ± 12 mg/g and KGF content of 18 ± 2 µg/g. Although the total protein content of GPE was significantly lower after gamma irradiation than before irradiation, the KGF content did not differ significantly between the non-irradiated and irradiated GPE. Moreover, the irradiated GPE complied with the microbiological quality requirements for microbial contamination (Tables S1 and S2). Figure 1 shows that GPE exhibited no cytotoxic effects on HGF cells, with cell viability remaining above 80% across the tested concentration range (1–4000 µg/mL). Notably, GPE significantly increased HGF cell viability at concentrations above 500 µg/mL (*p* < 0.05; Table S3), with maximal viability observed at 2000 µg/mL. GPE promoted HGF cell viability in a dose- and time-dependent manner, with concentrations of 500–2000 µg/mL significantly enhancing viability at 24, 48, and 72 h compared with untreated controls (*p* < 0.05). However, the present study was based on a single independent experiment; therefore, these findings should be considered preliminary and require confirmation using independent biological replicates.

### 3.2. Effect of GPE on Chemotherapy-Induced Gingival Fibroblast Cell Damage

**Figure 2:**
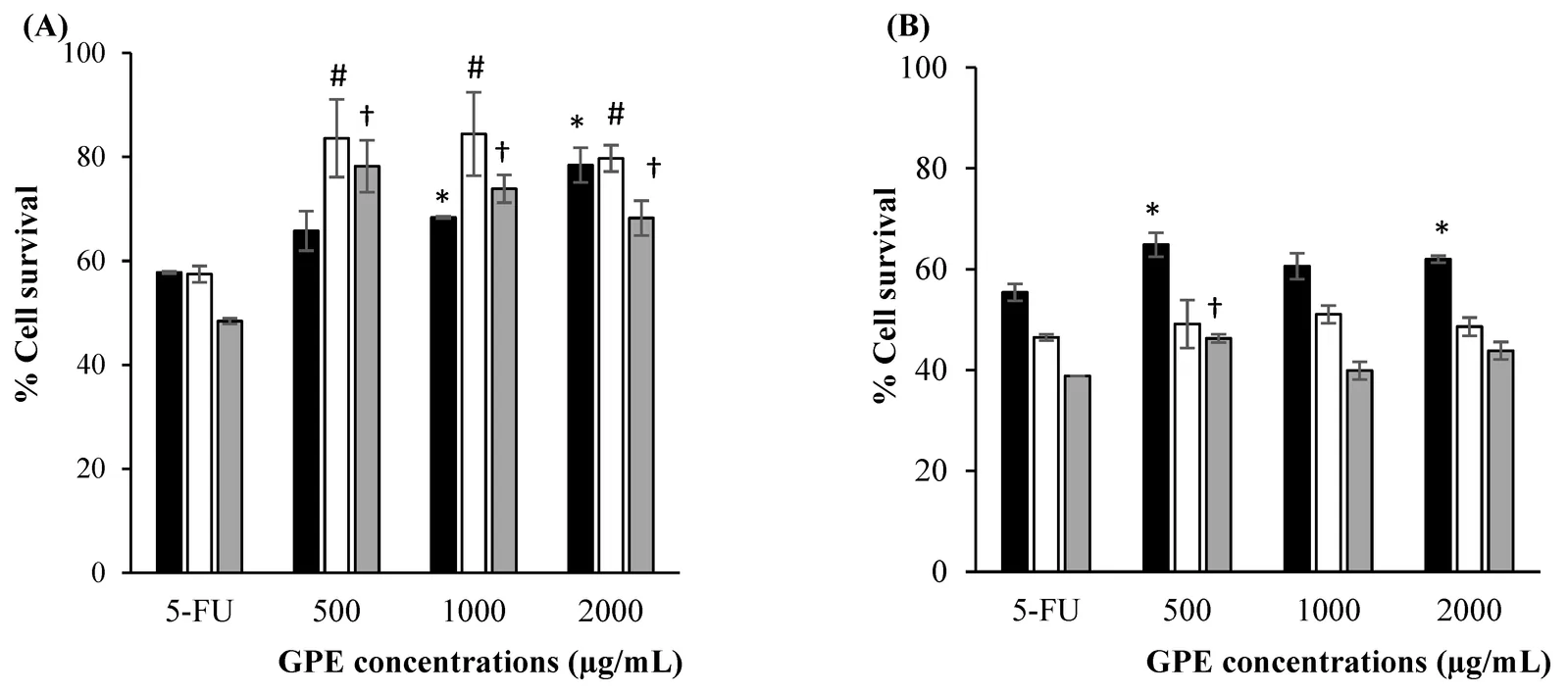
Percent cell survival of HGF cells treated with various concentrations of GPE. (**A**) Pre-treated cells with various concentrations of GPE before 5-FU exposure and (**B**) post-treated cells with various concentrations of GPE after 5-FU treatment. Data are expressed as mean ± SD. (*n* = 3). Asterisks indicate statistically significant differences from the 5-FU treatment, with * at 24 h (*p* < 0.05), # at 48 h (*p* < 0.05), and † at 72 h (*p* < 0.05).

For the cytoprotective effect in the chemotherapy-induced gingival fibroblast damage model (Figure 2), treatment with 5-FU resulted in a significant reduction in cell viability compared with untreated control cells, confirming successful induction of cytotoxic injury. Cell viability was suppressed to below 60% and 50% after 24 and 72 h of incubation, respectively. Pre-treatment with GPE (1000 and 2000 µg/mL) 24 h before 5-FU exposure significantly increased cell survival at 24 h and sustained cell metabolic activity at 48 and 72 h compared to 5-FU-treated cells. Similarly, pre-treated cells with 500 µg/mL GPE demonstrated recovery of cell viability at 48 and 72 h compared to the 5-FU-only treatment (*p* < 0.05) (Figure 2A). For the post-treatment group, GPE administration demonstrated a slightly elevated cell survival rate at 24 h and supported cell recovery compared to the 5-FU-treated-only group across all time intervals (Figure 2B). However, pre-treatment with GPE exerted a stronger cytoprotective effect than post-treatment. This suggests that GPE provided a protective effect in HGF cells, and pre-treatment provided superior protection compared to post-treatment.

**Figure 3:**
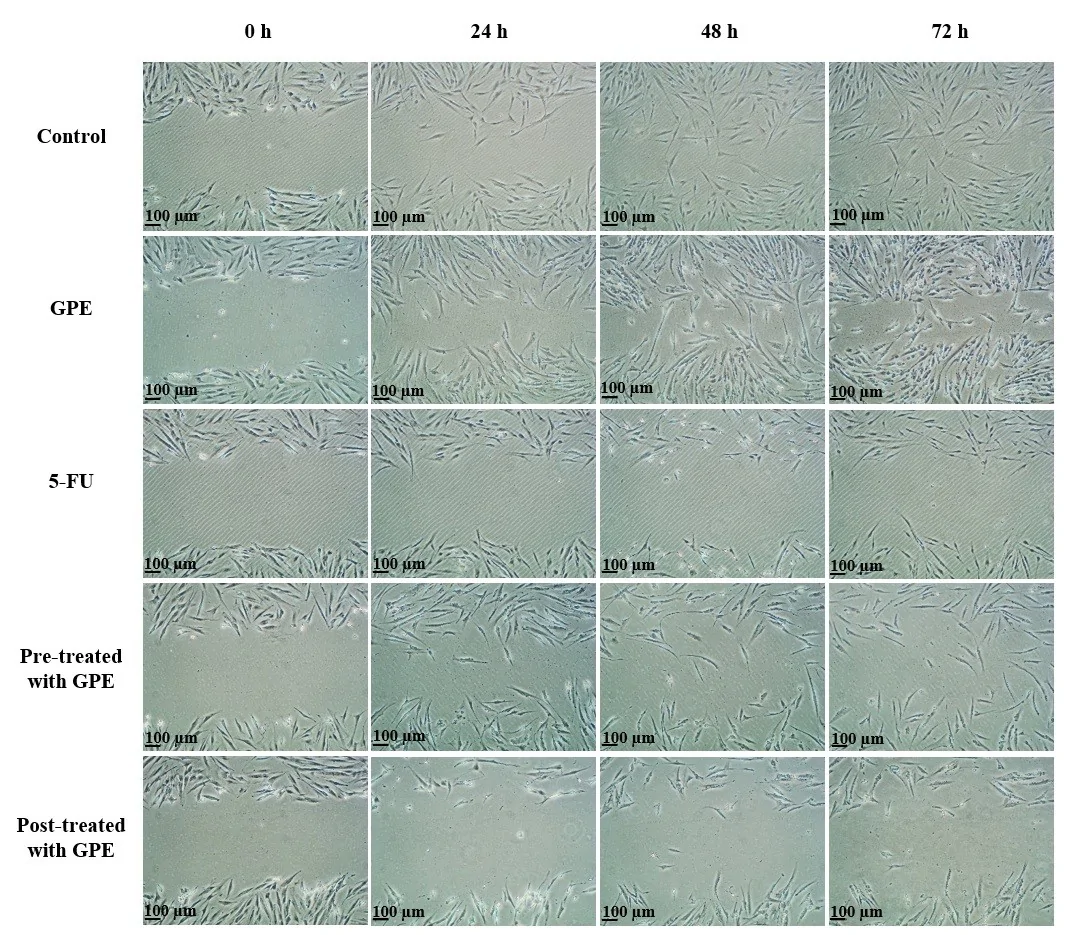
Images of wound closure after treatment with GPE (1000 µg/mL), 5-FU (10 mg/mL), pre-treated and post-treated with GPE in damaged cells, compared with a control (untreated cells) for 0, 24, 48, and 72 h (4× objective lens).

In the *in vitro* scratch assay (Figure 3), treatment with GPE at 1000 µg/mL increased gap closure compared with untreated control cells, indicating enhanced migratory activity of gingival fibroblasts. Specifically, after 48 and 72 h of treatment with GPE, the gap areas were markedly reduced compared to the control group. The 5-FU-treated cells exhibited impaired wound closure throughout the observation period. This finding indicates that GPE enhanced cellular wound healing responses in chemotherapy-damaged HGF cells through mechanisms that may include migration, recovery of cell viability, and restoration of cell function. Furthermore, pre-treatment with GPE prior to 5-FU exposure resulted in greater wound closure compared to post-treatment, suggesting that GPE exerts both protective and recovery-promoting effects against chemotherapy-induced cellular damage.

### 3.3. FTIR Spectrum of GPE-Loaded Dried Liposomes

**Figure 4:**
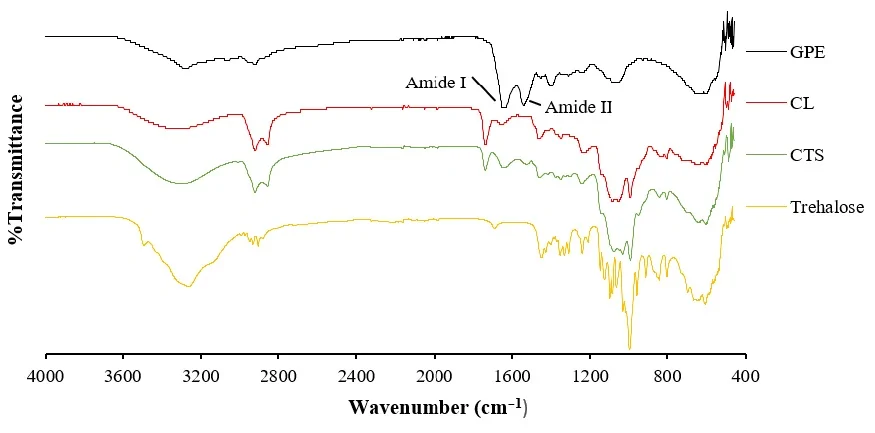
FTIR spectrum of GPE, GPE-loaded CL, GPE-loaded CTS, and trehalose.

The FTIR spectra of the GPE, GPE-loaded CL, GPE-loaded CTS, and trehalose are presented in Figure 4. GPE exhibited the most important spectral region of the amide I and II bands, indicating vibrations of the protein backbone. Amide I (C=O stretching; ~1610–1700 cm^−1^) is the most widely used spectral region for determining protein secondary structure. At lower wavenumbers, amide II (~1500–1570 cm^−1^) primarily arises from N-H bending coupled with C-N stretching vibrations [17].

The GPE-loaded liposomal formulations retained the amide I band, indicating the presence of protein-associated functional groups after formulation and lyophilization. Moreover, the spectra of liposomes showed characteristic phospholipid peaks at ~2926 and 2855 cm^−1^ relating to the asymmetric and symmetric stretching of C-H bonds within the fatty acid chain, ~1735 cm^−1^ relating to the C=O stretching in the fatty acid ester, ~1266 cm^−1^ relating to the P=O stretching, ~1163 cm^−1^ (P-O-C stretching), and ~968 cm^−1^ (associated with the asymmetric stretching of the -N^+^(CH_2_)_3_ group) [18]. For CTS, the increased intensity of amide I and II bands compared with unmodified liposomes can be attributed to the presence of chitosan on the vesicle surface [19]. Moreover, the cryoprotective agent, trehalose, in the liposomal formulation exhibited a low-intensity major peak. No new prominent spectral bands or substantial changes in the characteristic peaks were observed, suggesting that no major chemical interaction between trehalose and the liposomal components was evident from the FTIR spectra.

### 3.4. Characterization and Particle–Mucin Interaction of GPE-Loaded Liposomes

**Figure 5:**
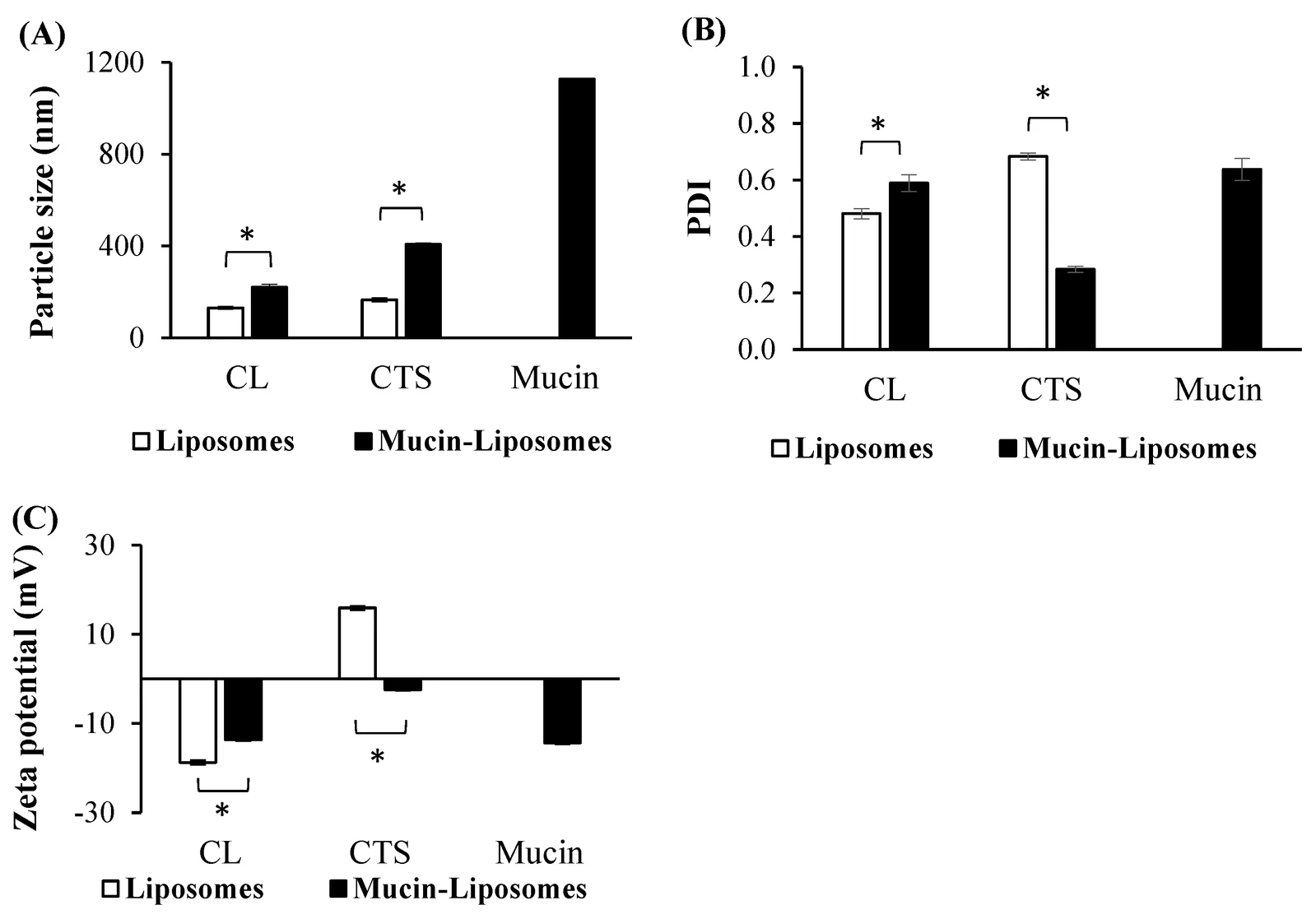
(**A**) Particle size, (**B**) polydispersity index (PDI), and (**C**) zeta potential of CL and CTS formulations after reconstitution in the absence and presence of mucin. Data are presented as mean ± SD (*n* = 3). * indicates a significant difference between samples with and without mucin interaction (*p* < 0.05).

The physicochemical properties of the GPE-loaded liposomes are presented in Figure 5. Both CL and CTS preserved nanometer-scale vesicles following the drying process (Table S4). The zeta potential remained negative for CL and positive for CTS, confirming the structural retention of lipid bilayers and chitosan coating after reconstitution. The apparent entrapment efficiency of protein from GPE was 29.9 ± 0.7% for CL and 33 ± 4% for CTS, indicating a slightly higher apparent protein entrapment in the chitosan-coated system. In this study, a 100-kDa centrifugal filter was used to separate the liposomal fraction from the unencapsulated fraction prior to protein quantification by the BCA assay. The use of centrifugal filtration for separating proteins from nanoparticle dispersions has been reported. Amicon Ultra centrifugal filters have been used to separate proteins, drugs, and nanoparticles, with protein passage depending on the characteristics of both the protein and the membrane. Although the marker protein standard sample (25–150 kDa) was applied to 100K filters and found to have filtered through the filter tube, free proteins larger than the membrane cut-off may also be retained [20]. In addition, the measured protein content in the formulation ranged from 101.31% to 110.69%. These values exceeding 100% may be associated with the complex protein composition of the crude GPE, in which small variations in the measured protein concentration and the retention of some free GPE proteins by the 100-kDa membrane filter may affect the calculated apparent entrapment efficiency and protein content.

The interaction between liposomal formulations and mucin was evaluated through measurements of particle size, PDI, and zeta potential (Figure 5). The mucin-only sample exhibited the largest hydrodynamic diameter, and the subsequent increase in liposomal size confirmed the formation of mucin–liposome aggregates or crosslinked structures. CTS showed a greater increase in size than CL did. The PDI of CL and CTS liposomes exhibited moderate size distribution, while exposure to mucin resulted in a broader size distribution. The lower PDI of CTS compared with CL after mucin interaction indicates that chitosan coating promotes more uniform mucin association. CTS initially exhibited a positive zeta potential, which markedly decreased upon mucin addition due to electrostatic neutralization between oppositely charged components, accompanied by an increase in particle size, confirming the formation of stable mucin–liposome complexes.

### 3.5. Characterization of the GPE-Loaded CTS-Oral Gel

The GPE-loaded CTS was homogeneously incorporated into the PVP K90 gel base, resulting in a smooth formulation without visible aggregation or coarse particles. The viscosity of the CTS-loaded oral gel was 4.5 ± 1.0 mPa·s, compared with 5.3 ± 1.1, 5.0 ± 0.7, and 0.0 ± 0.2 mPa·s for the commercial PVP gel, GPE gel, and CTS formulation, respectively. The gel formulations exhibited higher apparent viscosity than the corresponding solution formulations, reflecting the contribution of the gel matrix to the rheological properties of the formulations. However, the relatively low viscosity of the oral gel formulations is consistent with their intended use as mouth rinses, where low resistance to flow facilitates spreading throughout the oral cavity and allows the formulation to be readily rinsed and removed after use. The pH values of CTS and CTS gel were 5.34 ± 0.02 and 5.88 ± 0.02, respectively, indicating a mildly acidic environment that may be suitable for oral mucosal application.

### 3.6. Visualization of Buccal Penetration Pathways

**Figure 6:**
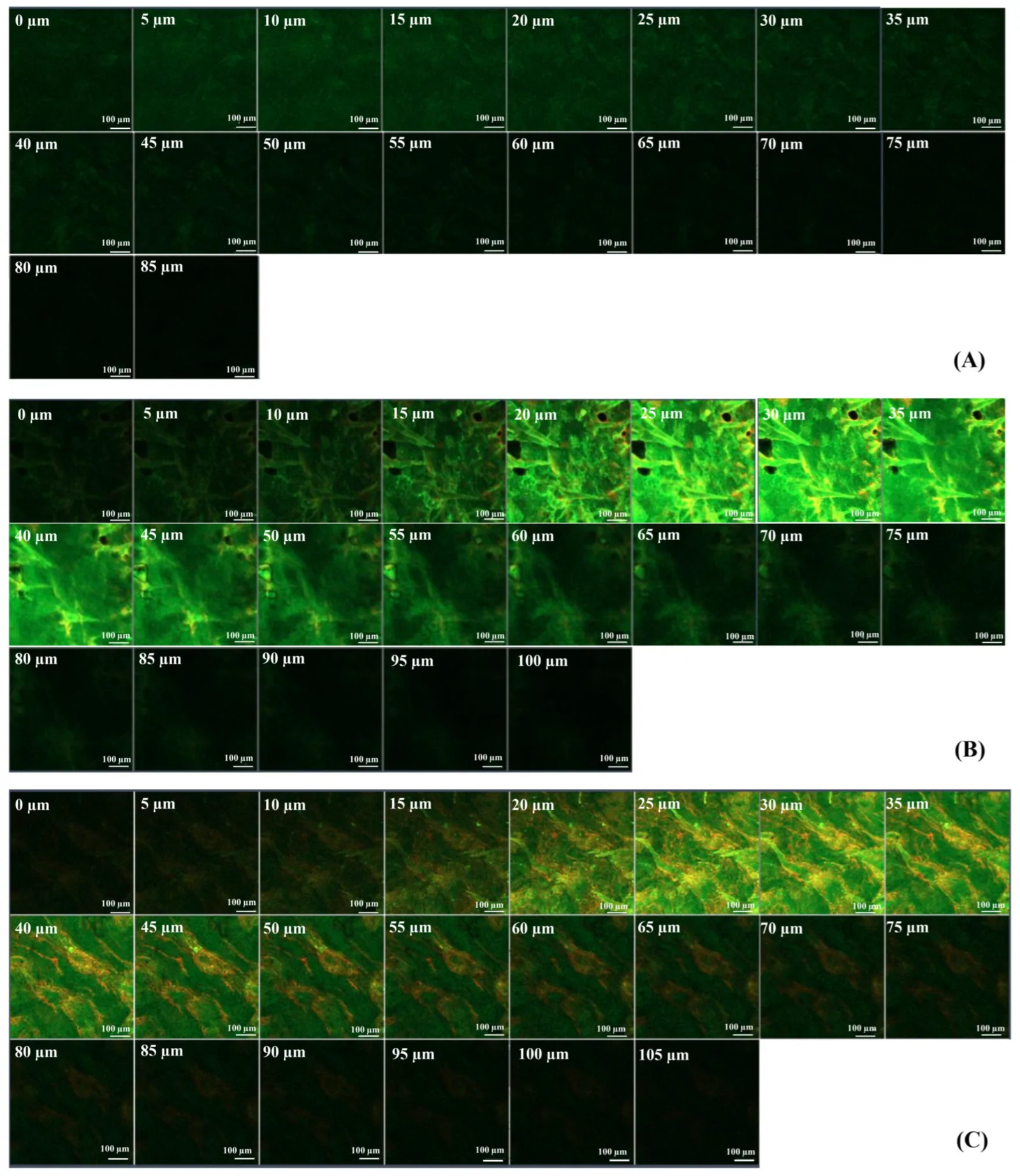
CLSM images (X-Y series) of buccal mucosa treated for 4 h with BSA-FITC gel (**A**), CTS (**B**), and CTS gel (**C**) (10 × objective lens). Green fluorescence represents BSA-FITC, while red fluorescence represents Rh-PE-labeled phospholipids.

To evaluate localized buccal mucosal deposition and penetration of the formulation, the distribution and penetration depth of the fluorescently labeled formulation within the buccal mucosa were visualized. Previous analysis showed that GPE contained a prominent protein band at approximately 66 kDa, similar to the molecular weight of albumin [6]. Because GPE itself does not exhibit detectable fluorescence, BSA-FITC was used as a fluorescent model macromolecular protein to visualize the distribution of the protein component within the buccal tissue. Rh-PE-labeled phospholipids were incorporated to visualize the nanocarriers [15]. BSA-FITC and Rh-PE exhibited green and red fluorescence, respectively (Figure 6). X-Y serial images of CTS and CTS gel exhibited the penetration of fluorescent compounds at depths of 100 μm and 105 μm, respectively, whereas the gel formulation demonstrated macromolecular protein distribution into the buccal mucosa at 85 μm depth. These findings indicate that CTS achieved greater localized penetration of the model macromolecular protein into the buccal mucosa than the conventional gel formulation.

**Figure 7:**
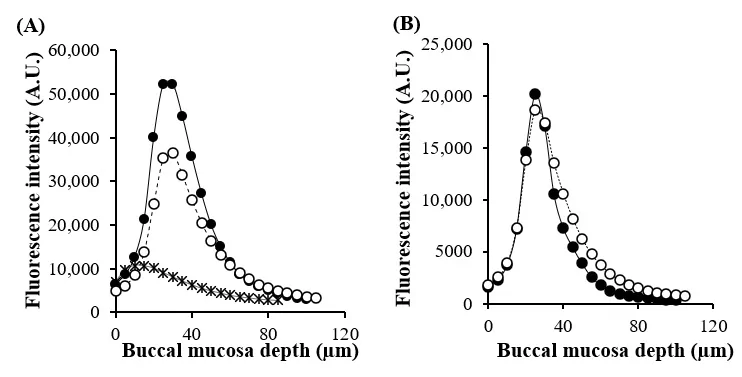
Fluorescence intensity profiles of (**A**) BSA-FITC and (**B**) Rh-PE-probed nanocarriers loaded with CTS (●), CTS gel (○), and gel (*) at different buccal mucosa depths.

The fluorescence intensity of CTS was greater than that of both the CTS gel and the gel formulation at all depths. For the CTS formulation, the fluorescence intensity of BSA-FITC reached a maximum at a depth of approximately 30 µm, while the fluorescence intensity of Rh-PE peaked at a depth of approximately 25 µm (Figure 7). These findings indicated the localized deposition of the model macromolecular protein and rhodamine conjugate phospholipid into the buccal mucosa.

## 4. Discussion

GPE contains bioactive macromolecular proteins and KGF, exhibiting a bioactivity effect on gingival fibroblasts. HGF cells represent the predominant stromal cells of the oral mucosa and are well known for their unique regenerative phenotype compared to skin fibroblasts and a greater capacity for scarless wound healing, largely mediated by differential growth factor release such as TGF-β, vascular endothelial growth factor-α (VEGF-α), and FGF-2 [21]. In addition, gingival fibroblasts contribute to immune regulation through CD14, TLR4, and MD-2 expression, alongside secretion of IL-6, a cytokine frequently used as a biomarker for stress and immune response in biocompatibility testing [22,23]. KGF, a bioactive component of GPE, has been evidenced to stimulate the cell cycle by enhancing cell cycle via G1/S transition via MEK/Erk pathways [24]. The ability of GPE to enhance the viability of HGF cells under non-cytotoxic conditions is consistent with established parameters of biological activity and indicates a potential role in modulating wound healing pathways.

The effects of GPE on cell viability may be attributed to its proteins and growth factor composition, particularly KGF, which supports epithelial repair. Comparable studies investigating dental biomaterials have emphasized the importance of bFGF and related growth factors in promoting cell proliferation, differentiation, and extracellular matrix production [25,26]. Thus, GPE not only sustains HGF viability but also enhances its regenerative capacity, underscoring its translational potential in oral tissue repair and as a therapeutic candidate for chemotherapy-induced oral mucosal damage.

5-FU is a commonly used chemotherapeutic agent that exerts antitumor activity primarily through the inhibition of DNA synthesis and cell proliferation, as well as the enhancement of cell death [27]. Nevertheless, its clinical use is frequently accompanied by oral mucositis, affecting approximately 20–50% of patients undergoing repeated cycles of chemotherapy [28]. The development of 5-FU-induced oral mucositis is largely attributed to increased inflammatory responses and cytotoxic effects on oral mucosal cells, particularly epithelial cells and fibroblasts [29]. Consistent with these mechanisms, the present study demonstrated reduced gingival fibroblast survival at 24–72 h and incomplete wound closure following 5-FU treatment.

Aqueous extracts of human placenta have been reported to possess anti-inflammatory and wound-healing properties [30], suggesting it as a beneficial therapeutic option for the management of chemo-radiation-induced acute oral mucositis [31]. Placental peptides can enhance epithelial cell proliferation and cell migration, thereby accelerating wound closure. These effects are associated with increased EGF secretion and downregulation of matrix metalloproteinase-2 (MMP-2) [32].

Currently, recombinant human KGF is one of the few available therapeutic options for oral mucositis, exerting protective effects on both keratinocytes and subepithelial cells. Its mechanism involves modulation of inflammatory cytokines, particularly interleukin-8 (IL-8) and tumor necrosis factor-α (TNF-α), leading to reduced tissue damage and enhanced recovery [33]. In the present study, GPE significantly enhanced HGF viability even under 5-FU-induced stress, indicating its potential to promote mucosal regeneration and wound healing. This cytoprotective effect is likely attributed to its bioactive components, including growth factors and essential nutrients, which support cellular repair and tissue regeneration [6]. Pre-treatment exhibited superior efficacy compared to post-treatment in both cell viability and wound healing. The greater effect observed with pre-treatment suggests that prior exposure to GPE may provide cellular protection against subsequent 5-FU-induced injury. Previous studies have suggested that growth factors, including KGF, effectively drive healthy fibroblasts through the G1/S phase transition via upstream signaling pathways. This proactive intervention upregulates the anti-apoptotic protein Bcl-2 [34]. However, these molecular mechanisms were not directly investigated in the present study and therefore remain hypothetical. Therefore, GPE represents a promising candidate for promoting the recovery of 5-FU-induced gingival fibroblast injury; however, further mechanistic studies are required to elucidate the molecular pathways underlying these effects.

The successful development of stable GPE-loaded CTS with mucin-interactive properties indicative of possible mucoadhesive behavior may support their potential suitability for buccal delivery of bioactive macromolecules. Protein–chitosan interactions are primarily governed by electrostatic forces, resulting in improved encapsulation efficiency and formulation stability [35]. The interaction of CTS with mucin can be explained by the electrostatic interactions between the positively charged chitosan coating and negatively charged mucin glycoproteins, resulting in mucin adsorption onto the vesicle surface [36]. Therefore, the changes in particle size and zeta potential after incubation with mucin indicate the mucoadhesive potential of the chitosan-coated vesicles.

Chitosan-coated nanoliposomes demonstrated an enhanced capacity to deliver bioactive macromolecules into the buccal mucosa. According to Sahatsapan et al. [12], chitosan–maleimide-coated liposomes exhibit pronounced mucoadhesive properties, prolonging residence time on mucosal surfaces and facilitating improved penetration into buccal tissue. These permeation-enhancing effects are primarily attributed to the cationic nature of chitosan, which promotes electrostatic interactions with negatively charged cell membranes. Such interactions induce a reversible reorganization of tight junction proteins, leading to transient opening of intercellular junctions, thereby enhancing drug permeation without compromising cell viability or membrane integrity. Furthermore, the permeation-enhancing effect of chitosan is synergistically supported by its intrinsic mucoadhesive properties, which extend drug residence time at the mucosal interface [37,38,39].

The freeze-drying process resulted in an increase in the particle size and PDI of CTS after reconstitution (Table S4), suggesting partial aggregation and/or structural rearrangement of the vesicles during the drying/reconstitution process. Freeze-drying, also known as lyophilization, is widely used to remove water and facilitate the long-term storage and handling of nanoparticle formulations [40]. The incorporation of cryoprotectants to embed nanocarriers within an amorphous matrix is a commonly used approach to reduce aggregation and facilitate the redispersion of lyophilized powders [41]. Trehalose has been investigated for its ability to protect liposomes against aggregation during freeze-drying [42]; however, the addition and concentration of trehalose may also influence the physicochemical properties of liposomes after reconstitution, including particle size and PDI [43]. In the present study, although the particle size and PDI increased after freeze-drying and reconstitution, the formulation retained a nanosized particle population. Furthermore, the reconstituted CTS was compatible with subsequent incorporation into a mucoadhesive gel, supporting its potential use as a solid intermediate for a gel-based buccal delivery system.

The GPE-loaded CTS gel was successfully formulated and effectively suspended macromolecules and nanovesicles. PVP is a biocompatible mucoadhesive polymer in gel-based formulations designed for topical oral application, particularly for alleviating pain associated with oral mucositis and other mucosal lesions [44]. PVP-based hydrogels have also demonstrated sustained drug release behavior [45]. Therefore, the incorporation of chitosan-coated nanoliposomes into a PVP-based gel matrix may provide a potentially useful platform for localized buccal delivery. The observed mucosal penetration and distribution of the model macromolecular protein support the potential application of this platform for the localized treatment and regeneration of chemotherapy-induced oral mucositis. Nevertheless, additional *in vivo* and clinical studies are required to confirm its therapeutic efficacy, mucosal safety, local tolerability, and clinical applicability. Further studies are also required to evaluate the long-term stability of the formulation and to establish the release and biological activity of the encapsulated bioactive macromolecules under relevant conditions.

## 5. Conclusions

GPE contains bioactive macromolecules, including proteins and growth factors (KGF), which stimulate gingival fibroblast viability and contribute to the prevention and repair of chemotherapy-induced cellular damage. The GPE-loaded CTS formulation exhibited suitable physicochemical properties and potential mucoadhesive ability. Fluorescence imaging demonstrated localized deposition of the model macromolecular protein and rhodamine conjugate phospholipid into the buccal mucosa for both CTS and the CTS-loaded oral gel. These findings support the potential of the formulation for buccal application of bioactive macromolecular protein. However, further studies using more comprehensive models are warranted to determine its potential application in the supportive management of chemotherapy-induced oral mucosal injury.

## Figures and Tables

**Table 1:** The composition of liposomal formulations (%).

Formulations	PC	Cholesterol	GPE	Polysorbate 20	Water	Chitosan	1% HCl
CL	0.77	0.08	0.2	2	96.95	-	-
CTS	0.77	0.08	0.2	2	86.95	0.8	9.2

## Data Availability

The original contributions presented in this study are included in the article/Supplementary Materials. Further inquiries can be directed to the corresponding author.

## Supplementary Materials

The following supporting information can be downloaded at https://www.mdpi.com/article/10.3390/pharmaceutics18101246/s1, Figure S1: Percent cell viability of HGF cells treated with various concentrations of 5-FU. Data are presented as mean ± SD (*n* = 3); Figure S2: Shear stress and apparent viscosity versus shear rate of GPE-loaded CTS gel; Table S1: Total protein and KGF contents in GPE before and after gamma irradiation; Table S2: Specification and certificate of analysis of GPE; Table S3: The significant values of cell viability and cell survival of HGF cells treated with various concentrations of GPE; Table S4: Particle size, PDI, and zeta potential of liposomes before and after the drying process.

## Abbreviations

The following abbreviations are used in this manuscript:

%EE	Percent entrapment efficiency
5-FU	5-fluorouracil
ANOVA	Analysis of variance
ATR-FTIR	Attenuated total reflection–Fourier-transform infrared spectroscopy
BCA	Bicinchoninic acid
BSA-FITC	Bovine serum albumin–fluorescein isothiocyanate conjugate
CL	Conventional liposomes
CLSM	Confocal laser scanning microscopy
CTS	Chitosome
DLS	Dynamic light scattering
DMEM	Dulbecco’s modified Eagle’s medium
DMSO	Dimethyl sulfoxide
EGF	Epidermal growth factor
ELISA	Enzyme-linked immunosorbent assay
FBS	Fetal bovine serum
FGF	Fibroblast growth factor
GPE	Goat placenta extract
HGFs	Human gingival fibroblasts
IGF-1	Insulin-like growth factor-1
IL-8	Interleukin-8
KGF	Keratinocyte growth factor
MMP-2	Matrix metalloproteinase-2
MTT	3-(4,5-Dimethylthiazol-2-yl)-2,5-diphenyltetrazolium bromide
PBS	Phosphate-buffered saline
PC	Phosphatidylcholine
PDI	Polydispersity index
PVP	Polyvinylpyrrolidone
Rh-PE	Rhodamine B 1,2-dihexadecanoyl-sn-glycero-3-phosphoethanolamine triethylammonium salt
SD	Standard deviation
TGF- β1	Transforming growth factor-β1
TNF-α	Tumor necrosis factor-α
Trypsin–EDTA	Trypsin–ethylenediaminetetraacetic acid
VEGF-α	Vascular endothelial growth factor-α
