# Supplementary material for: Mucoadhesive Chitosome-Based Buccal Delivery of Goat Placenta-Derived Bioactive Macromolecules: Effects on 5-Fluorouracil-Induced Gingival Fibroblast Injury
Source: Pharmaceutics. 2026 Oct 1;18(10):1246. doi: 10.3390/pharmaceutics18101246 (PMC13708512; doi:10.3390/pharmaceutics18101246)
Supplement: Supplementary file 1 [file pharmaceutics-18-01246-s001.zip › pharmaceutics-4532984-supplementary.pdf]

Supplementary file

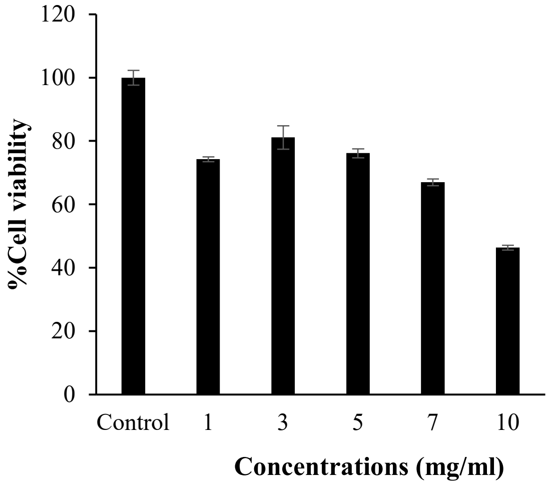

Figure S1: Percent cell viability of HGF cells treated with various concentrations of 5-FU. Data are presented as mean ± SD (n = 3).

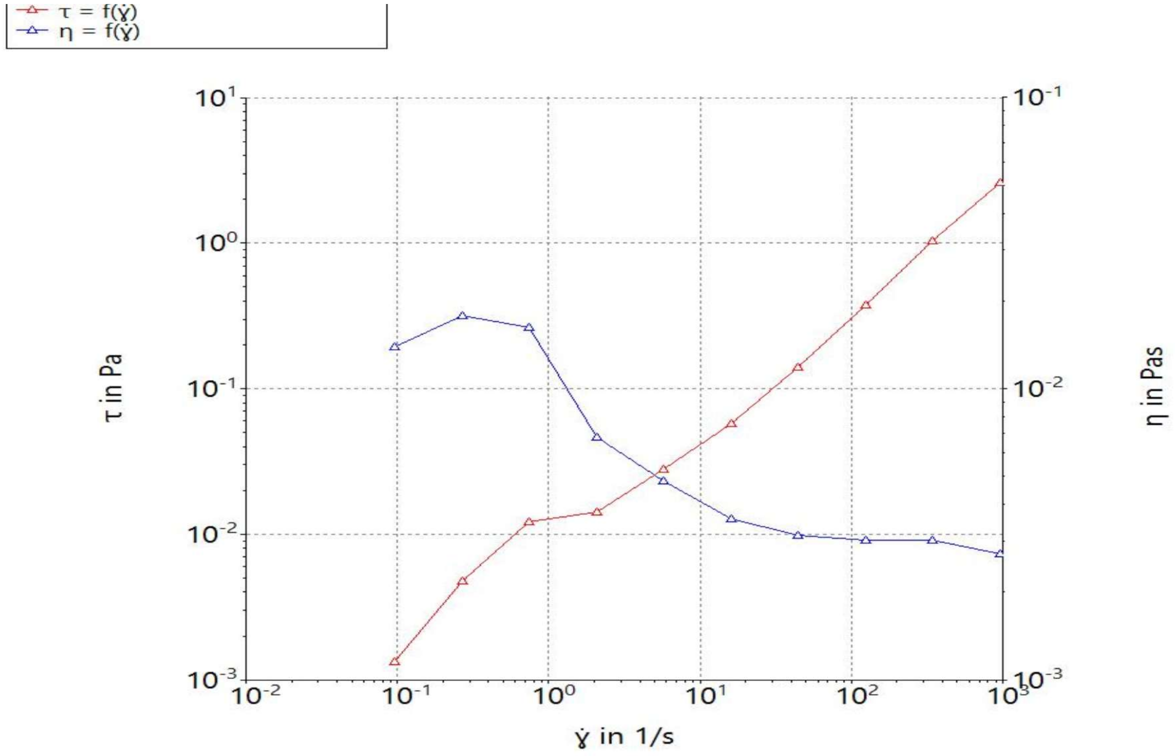

Figure S2: Shear stress and apparent viscosity versus shear rate of GPE-loaded CTS gel

Table S1: Total protein and KGF contents in GPE before and after gamma irradiation

Samples	Total protein content (mg/g)	KGF content (μg/g)
GPE	652 ± 8	16 ± 5
Gamma-irradiated GPE	544 ± 12*	18 ± 2

Data represent means ± SD (n=3). * indicates significant differences from non-irradiated GPE ($p < 0.05$).

Table S2: Specification and certificate of analysis of GPE

**Product Name**	Goat Placenta Extract	**Latin Name**	*Capra aegagrus hircus*
**Part Used**	Placenta	**Batch No.**	F001/67
**Solvent Residual**	Water	**Manufacture Date**	11 June 2024
**Quantity**	9.3 g	**Expiry Date**	10 June 2026

ITEMS	SPECIFICATIONS	RESULTS	METHODS
Appearance	brownish-red, fibrous-textured powder	Complies	Visual
Odor	Characteristic	Complies	Organoleptic
Solubility	Freely soluble (≤ 200 mg/mL)	Complies	Water solubility test
Moisture content	≤ 10.0%	6.6%	Loss on drying
Protein	≥50.0%	54.4%	BCA protein assay
pH (0.1 g/mL aqueous solution)	5.0-7.5	6.0	pH meter
**Heavy metals**			
Arsenic (As)	≤ 5 ppm	Not detected	Atomic Emission Method
Lead (Pb)	≤ 10 ppm	Not detected	Atomic Emission Method
Cadmium (Cd)	≤ 0.3 ppm	Not detected	Atomic Emission Method
Mercury (Hg)	≤ 0.5 ppm	Not detected	Atomic Emission Method
**Microbiological**			
Total aerobic microbial count	< 5 x 10^4 CFU/g	Complies	Thai herbal Pharmacopeia
Staphylococcus aureus	Not detected in 1 g	Not detected	Thai herbal Pharmacopeia

Salmonella spp.	Not detected in 25 g	Not detected	Thai herbal Pharmacopeia
Escherichia coli	Not detected in 1 g	Not detected	Thai herbal Pharmacopeia
Clostridium perfringens	Not detected in 1 g	Not detected	Thai herbal Pharmacopeia
Packaging and storage	Packed in a well-closed container. Keep in a cool and dry place.	Complies	

Table S3: The significant values of cell viability and cell survival of HGF cells treated with various concentrations of GPE

Tests	Concentrations (μg/mL)	Sig.
**Cell viability (10^4 cell/well)**		
Control **at 24 h**	1	1.00
	10	1.00
	100	< 0.001
	500	< 0.001
	1000	< 0.001
	2000	< 0.001
	4000	< 0.001
**Cell viability ($5x10^3$ cell/well)**		
Control at 24 h	500	< 0.001
	1000	< 0.001
	2000	< 0.001
Control at 48 h	500	< 0.001
	1000	< 0.001
	2000	< 0.001
Control at 72 h	500	< 0.001
	1000	< 0.001
	2000	< 0.001
**Cell survival**		
**Pre-treated cells**		
5-FU at 24 h	500	0.06
	1000	< 0.001
	2000	< 0.001
5-FU at 48 h	500	< 0.001
	1000	< 0.001

	2000	< 0.001
5-FU at 72 h	500	< 0.001
	1000	< 0.001
	2000	< 0.001
**Post-treated cells**		
5-FU at 24 h	500	<0.001
	1000	0.08
	2000	0.02
5-FU at 48 h	500	0.68
	1000	0.22
	2000	0.82
5-FU at 72 h	500	0.01
	1000	0.97
	2000	0.08

Table S4. Particle size, PDI, and zeta potential of liposomes before and after the drying process.

Formulations	Size (nm)	PDI	Zeta potential (mV)
**Liposomes**			
CL	67 ± 1	0.31 ± 0.01	−13 ± 1
CTS	73.0 ± 0.5	0.23 ± 0.01	+17 ± 4
**Dry Liposomes**			
CL	130 ± 3	0.48 ± 0.02	−18.7 ± 0.4
CTS	165 ± 5	0.68 ± 0.01	+15.9 ± 0.4

Data represent means ± SD (n=3).
